# Mapping of ESE-1 subdomains required to initiate mammary epithelial cell transformation via a cytoplasmic mechanism

**DOI:** 10.1186/1476-4598-10-103

**Published:** 2011-08-28

**Authors:** Jason D Prescott, Joanna M Poczobutt, John J Tentler, Darius M Walker, Arthur Gutierrez-Hartmann

**Affiliations:** 1 Medical Scientist Training Program, University of Colorado Denver, Aurora, CO 80045, USA; 2 Program in Molecular Biology, University of Colorado Denver, Aurora, CO 80045, USA; 3 Department of Medicine, University of Colorado Denver, Aurora, CO 80045, USA; 4 Department Biochemistry & Molecular Genetics, University of Colorado Denver, Aurora, CO 80045, USA

## Abstract

**Background:**

The ETS family transcription factor ESE-1 is often overexpressed in human breast cancer. ESE-1 initiates transformation of MCF-12A cells via a non-transcriptional, cytoplasmic process that is mediated by a unique 40-amino acid serine and aspartic acid rich (SAR) subdomain, whereas, ESE-1's nuclear transcriptional property is required to maintain the transformed phenotype of MCF7, ZR-75-1 and T47D breast cancer cells.

**Results:**

To map the minimal functional nuclear localization (NLS) and nuclear export (NES) signals, we fused in-frame putative NLS and NES motifs between GFP and the SAR domain. Using these GFP constructs as reporters of subcellular localization, we mapped a single NLS to six basic amino acids (^242^HGKRRR^247^) in the AT-hook and two CRM1-dependent NES motifs, one to the pointed domain (NES1: ^102^LCNCALEELRL^112^) and another to the DNA binding domain (DBD), (NES2: ^275^LWEFIRDILI^284^). Moreover, analysis of a putative NLS located in the DBD (^316^GQKKKNSN^323^) by a similar GFP-SAR reporter or by internal deletion of the DBD, revealed this sequence to lack NLS activity. To assess the role of NES2 in regulating ESE-1 subcellular localization and subsequent transformation potency, we site-specifically mutagenized NES2, within full-length GFP-ESE-1 and GFP-NES2-SAR reporter constructs. These studies show that site-specific mutation of NES2 completely abrogates ESE-1 transforming activity. Furthermore, we show that exclusive cytoplasmic targeting of the SAR domain is sufficient to initiate transformation, and we report that an intact SAR domain is required, since block mutagenesis reveals that an intact SAR domain is necessary to maintain its full transforming potency. Finally, using a monoclonal antibody targeting the SAR domain, we demonstrate that the SAR domain contains a region accessible for protein - protein interactions.

**Conclusions:**

These data highlight that ESE-1 contains NLS and NES signals that play a critical role in regulating its subcellular localization and function, and that an intact SAR domain mediates MEC transformation exclusively in the cytoplasm, via a novel nontranscriptional mechanism, whereby the SAR motif is accessible for ligand and/or protein interactions. These findings are significant, since they provide novel molecular insights into the functions of ETS transcription factors in mammary cell transformation.

## Background

The human ETS (E26 Transformation-Specific) protein family is a diverse group of 27 known transcription factors that regulate such varied cellular processes as differentiation and apoptosis, but also appear to induce oncogenesis when mutated or aberrantly expressed [1-4]. In particular, aberrant ETS protein activity and/or expression has been implicated in human mammary epithelial cell (MEC) transformation [5]. The ER81 ETS protein, for example, is activated in human breast cancer cells by the oncoprotein HER-2, resulting in over-expression of the prosurvival telomerase reverse transcriptase (hTERT) gene [6]. In addition, ETS-1 mRNA overexpression appears to be a strong independent predictor of poor prognosis in primary human breast cancers [7]. Furthermore, ETS-2 overexpression can inhibit expression of the tumor-suppressor gene BRCA1, the downregulation of which is clearly linked to familial breast cancer [7,8].

Overexpression of one ETS protein in particular, the epithelium-specific ETS factor ESE-1, is implicated in human mammary transformation. ESE-1 mRNA is overexpressed in primary human ductal carcinomas *in situ *(DCIS), and the genomic ESE-1 locus (1q32.1) is commonly amplified in primary human breast cancer cells [9-11]. In addition, we have shown that ESE-1 expression confers a transformed phenotype to the nontransformed MCF-12A and MCF-10A human MECs, including enhanced invasiveness and motility, anchorage independent growth, epidermal growth factor-independent proliferation, and formation of disorganized structures in three-dimensional cultures on matrigel [12-14]. A later study screening a collection cDNAs associated with breast cancer independently identified ESE-1 as a factor that promotes motility and induces formation of disorganized structures on matrigel in MCF-10A cells [15].

While previous publications have established ESE-1's transcription factor function, we have reported that ESE-1 initiates transformation of MECs via a novel non-nuclear, non-transcriptional mechanism [13]. We have shown that a 40-amino acid (AA) serine and aspartic acid rich (SAR) domain within the ESE-1 is both necessary and sufficient to mediate ESE-1 transforming function and that enforced nuclear localization of full-length ESE-1 or of the SAR domain alone, abrogates ESE-1 ability to initiate transformation [13]. These results imply that ESE-1 contains an endogenous nuclear export signal that is required for ESE-1-mediated initiation of MEC transformation via a cytoplasmic mechanism. In addition to transformation initiating function that requires cytoplasmic localization of ESE-1, we have reported that ESE-1 is required for the maintenance of transformed phenotype in breast cancer cell lines. We have shown that shRNA-mediated downregulation of ESE-1 protein levels in MCF7 and ZR-75-1 breast cancer cell lines results in decreased anchorage independent growth, and that in these cells lines, as well as in T47D, ESE-1 is localized to the nucleus [16]. Thus, nuclear function of ESE-1 is required for the maintenance of transformed phenotype. Together these reports establish that nuclear-cytoplasmic shuttling of ESE-1 is essential for transformation initiation in benign MECs as well as for the maintenance of transformed phenotype in breast cancer cells, and imply that ESE-1 contains both nuclear export (NES) as well as nuclear localization (NLS) signals.

In the current report we use fusion between green fluorescent protein (GFP) and specific ESE-1 motifs to map functional ESE-1 NES and NLS sequences and to define the role of these motifs in ESE-1 transforming function. We localize the functional ESE-1 NLS to a six AA basic motif within the ESE-1 A/T Hook domain and we demonstrate that, unlike in other ETS proteins [17-19], in-frame deletion of the ESE-1 DBD does not abrogate ESE-1 nuclear localization. Using both gain-of-function and loss-of-function approaches, we identify a single NES within the ESE-1 DBD that is required for ESE-1-mediated initiation of MCF-12A cell transformation. Furthermore, we sequentially mutagenize 11-14 AAs blocks in the SAR domain to establish that while each of the SAR mutants partially retains transformation function in MCF-12A cells, an intact SAR domain is required for its full transforming activity. Finally, we identify ESE-1 region 216-228 within the SAR domain as the site of interaction with anti-ESE-1 antibody mAB405, what suggests that this region is surface exposed and thus likely to mediate protein-protein interactions. In summary, these data represent a paradigm shift in our understanding of the specific subcellular functions of ETS transcription factors, by revealing a novel NES2 and providing insights into SAR domain-dependent cytoplasmic mechanism by which ESE-1 initiates MEC transformation.

## Results

### ESE-1 contains a single, basic amino acid-rich NLS that maps to the A/T hook domain

**Figure 1:**
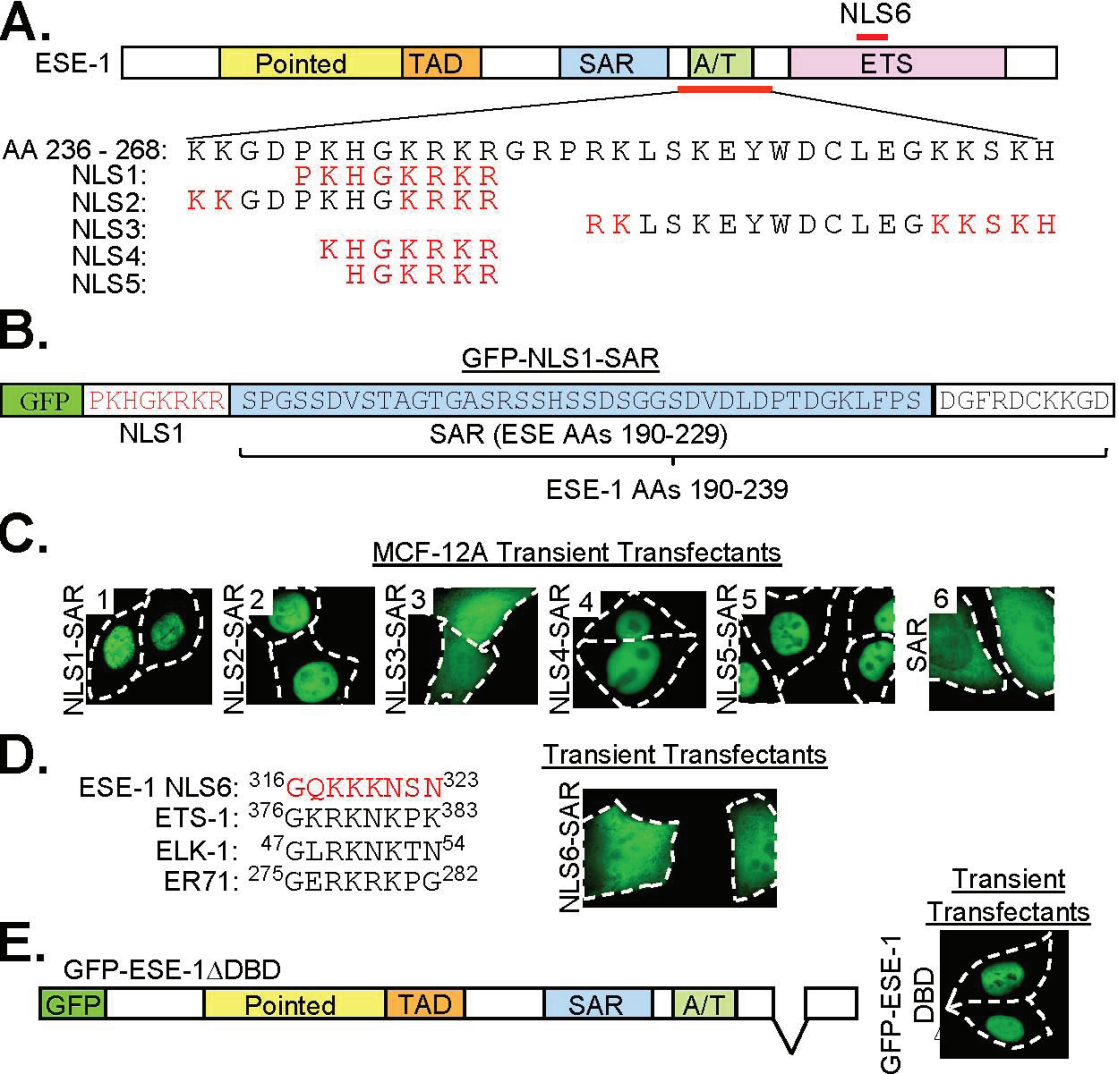
**ESE-1 contains a basic AA-rich NLS sequence**. **(A) **Diagram of ESE-1 protein and sequence of the ESE-1 region containing putative ESE-1 NLS motifs 1-5 (AAs 236-268). Colored boxes indicate ESE-1 domains. A red line below the diagram indicates localization of AAs 236-268, with the sequence expanded below. AA sequences for putative ESE-1 NLS motifs 1-5 are aligned with the ESE-1 236-268 AA sequence. Red letters - AAs potentially required for nuclear localization. Red line above diagram - localization of putative NLS6 motif. **(B) **Map and AA sequence of GFP-NLS1-SAR. GFP (green box, not to scale) followed in-frame by NLS1 (red letters). The AA sequence for all but the first two amino acids in the ESE-1 SAR domain (blue box) is fused to the carboxy-terminal end of NLS1, followed by ESE-1 flanking sequence (white box). All remaining NLS fusion proteins were constructed the same way. **(C) **Representative fluorescence microscopy images of MCF-12A cells transiently transfected with GFP-NLS1-SAR (panel 1), GFP-NLS2-SAR (panel 2), GFP-NLS3-SAR (panel 3), GFP-NLS4-SAR (panel 4), GFP-NLS5-SAR (panel 5), or GFP-SAR (panel 6) **(D) **Left panel - AA sequence alignments among putative ESE-1 NLS6 and ETS-1, ELK-1, ER71 NLS motifs. Superscripted numbering indicates the position of each AA sequence within its parental protein. Right panel - representative fluorescence microscopy images of MCF-12A cells, transiently transfected with the GFP-NLS6-SAR. **(E) **Left panel - diagram of GFP-ESE-1ΔDBD. In-frame deletion of ESE-1 DBD is represented by a V-shaped line. Right panel - representative MCF-12A cells transiently transfected with the GFP-ESE-1ΔDBD construct imaged by fluorescence microscopy. In all images white dashed lines represent cell outlines.

The nuclear localization signal (NLS) in Elf3, the murine ortholog of human ESE-1, has been mapped to four basic residues ^244^KRKR^247 ^within the AT Hook domain [20], with additional NLS motifs within the AT hook (AA 236-252 and 249-267) and DBD (^318^KKK^320^) also contributing to nuclear localization [21]. We have previously shown that in-frame deletion of AA 231-268, spanning the AT hook domain in human ESE-1, resulted in exclusive cytoplasmic localization [13]. To precisely map the functional NLS motif(s) within human ESE-1 and to assess whether these motif(s) are the same as in murine Elf3, we designed a gain-of-function assay, in which each putative NLS was fused between the GFP and SAR portions of the GFP-SAR construct [13] and the resulting GFP signals were then used as reporters of the subcellular localization of each fusion protein in transiently transfected MCF-12A cells (Figure 1). We identified one putative SV40-like NLS (NLS1: ^240^PKHGKRKR^249^), and two putative bipartite NLS sequences (NLS2: ^236^KKGDPKHGKRKR^247 ^and NLS3, ^251^RKLSKEYWDCLEGKKSKH^268^) (Figure 1A), which were also in murine Elf3 [21]. Subsequently, we generated GFP-NLS1-SAR, GFP-NLS2-SAR, and GFP-NLS3-SAR fusion constructs, in which each putative NLS was fused in-frame between GFP and a 189-239 AA fragment of ESE-1 spanning the SAR domain and 10 AAs just distal to the SAR domain (Figure 1A &1B). In transiently transfected MCF-12A cells, GFP-SAR protein is distributed to both the nuclear and cytoplasmic compartments (Figure 1C, panel 6) and [13]. In contrast, MCF-12A cells transiently transfected with the NLS fusion constructs demonstrate exclusive nuclear localization of GFP-NLS1-SAR (Figure 1C, panel 1) and GFP-NLS2-SAR (Figure 1C, panel 2). Whereas, GFP-NLS3-SAR is diffusely cytoplasmic and nuclear (Figure 1C, panel 3) and is indistinguishable from GFP-SAR protein (Figure 1C, panel 6). Thus, NLS1 and NLS2, but not NLS3, have intrinsic nuclear localization function, narrowing ESE-1 nuclear localizing activity to AA 236-249 (Figure 1A). To further localize ESE-1 NLS activity, two plasmids with progressive amino-terminal truncations of the ESE-1 NLS region were generated: pEGFP-NLS4-SAR and pEGFP-NLS5-SAR, in which the ESE-1 sequences ^241^KHGKRKR^247 ^and ^242^HGKRKR^247 ^were fused between GFP and SAR, respectively (Figures 1A &1B). Transient expression of both GFP-NLS4-SAR (Figure 1C, panel 4) and GFP-NLS5-SAR (Figure 1C, panel 5) demonstrated exclusive nuclear localization in MCF-12A cells. Taken together, these findings map more precisely ESE-1 nuclear localizing activity to AA 242-247 and define the ESE-1 NLS as a six amino acid sequence similar to the SV40 large T antigen NLS (PKKKRKV) [22].

Previous reports have shown that basic AA-rich sequences in the DBDs of several different ETS proteins, including ETS-1 (^376^GKRKNKPK^383^), ELK-1 (^47^GLRKNKTN^54^), and ER71 (^275^GERKRKPG^282^), mediate the nuclear localization of these proteins [17-19]. In murine Elf3, internal deletion or site-specific mutation of the ^318^KKK^320 ^sequence, within the context of the entire DBD, resulted in the localization to both the nucleus and the cytoplasm [21]. Considering these data, we tested whether a similar putative NLS sequence, ^316^GQKKKNSN^323 ^(ESE-1 NLS6) in the ESE-1 DBD also shows NLS function, using the GFP fusion strategy described above (Figure 1D). Transient expression of GFP-NLS6-SAR in MCF-12A cells revealed diffuse cytoplasmic and nuclear fluorescence (Figure 1D) that was indistinguishable from that of GFP-SAR (Figure 1C, panel 6) and [13], indicating that ESE-1 NLS6 is insufficient to mediate nuclear localization. To test whether the ESE-1 NLS6 is necessary to mediate nuclear localization, we generated an additional construct in which the ESE-1 DBD was deleted in-frame from the previously described pEGFP-ESE-1 expression plasmid, containing the full-length ESE-1 protein, to generate pEGFP-ESE-1ΔDBD (Figure 1E). Transient transfection in MCF-12A cells revealed exclusive nuclear GFP-ESE-1ΔDBD localization, thus demonstrating that in the human ortholog of ESE-1, the DBD is not required for ESE-1 nuclear localization. Together with the data shown in Figures 1C & Figure 1D, these findings indicate that, unlike previously examined ETS proteins, the ETS DBD does not play a role in ESE-1 nuclear localization.

### ESE-1 contains two separate CRM1-dependent NES motifs

**Figure 2:**
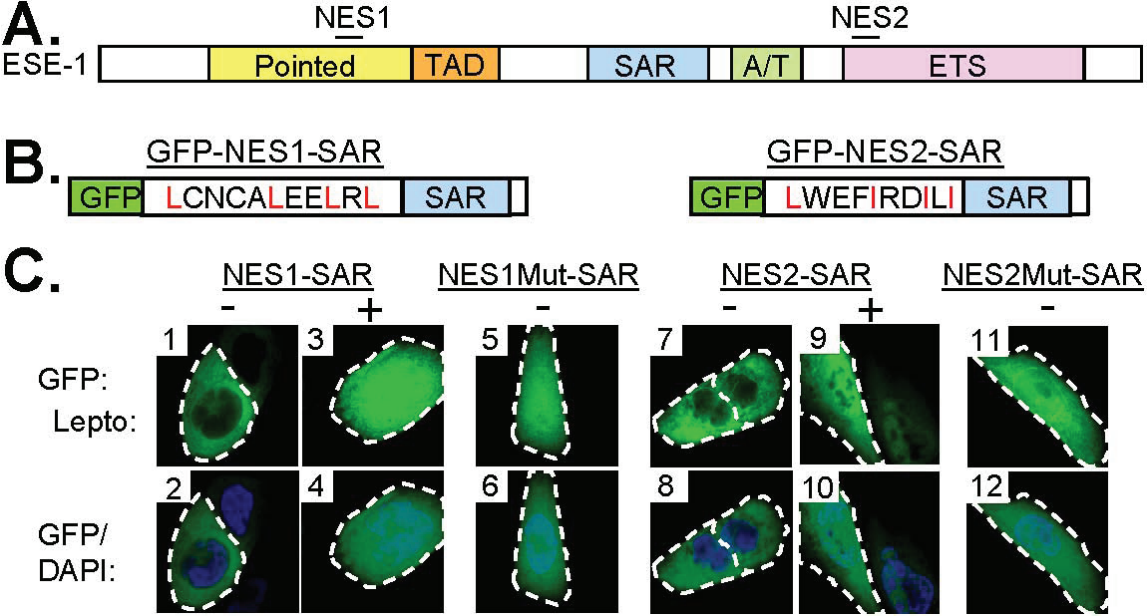
**ESE-1 contains two separate CRM-1 dependent NES motifs**. **(A) **Block diagram of ESE-1 protein with domains indicated by labeled colored boxes. NES1 and NES2 are represented by black lines above the diagram. **(B) **Schematic of GFP-NES1-SAR and GFP-NES2-SAR proteins, constructed as described in Figure 1B. Green box - GFP. White box - NES1 or NES2. Critical NES leucine and isoleucine residues are shown in red. Blue box followed by white box - ESE-1 fragment 190-239 containing the SAR domain (AAs 190-229). **(C) **Fluorescence imaging of transiently expressed GFP-NES1-SAR (± leptomycin B; panels 1, 2, 3, & 4), GFP-NES1Mut-SAR (panels 5 and 6), GFP-NES-2-SAR (± leptomycin B; panels 7, 8, 9, & 10), and GFP-NES2Mut-SAR (panels 11 & 12) in living MCF-12A cells. All transfectants were DAPI-stained to identify the nuclei and some GFP-NES1-SAR and GFP-NES2-SAR transfectants were treated with leptomycin B prior to microscopy. Upper panels - GFP fluorescence (green). Lower panels - overlays of GFP (green) and DAPI (blue) fluorescence. Although only one or two representative cells are shown for each transfectant population, a ~30% transfection efficiency was consistently achieved per 10^6 ^cells transfected and ~10^5 ^green fluorescent cells were present following each transfection. White dashed lines represent cell outlines.

Having shown that internal deletion of the AT hook domain containing the functional NLS results in exclusive cytoplasmic localization of ESE-1 [13], we speculated that ESE-1 contains two putative NES signals corresponding to the consensus sequence (L/I)X2-4(L/I)X1-4(L/I)X(L/I) [23]: ^102^LCNCALEELRL^112 ^in the Pointed domain (NES1) and ^275^LWEFIRDILI^284 ^in the DBD (NES2) (Figure 2A). To test the function of these NES motifs, we inserted each sequence in-frame between the GFP and SAR portions of the GFP-SAR construct to produce GFP-NES1-SAR and GFP-NES2-SAR, respectively (Figure 2B) and we used the GFP fluorescence as a reporter of subcellular localization. MCF-12A cells transiently transfected with these constructs demonstrate a predominantly cytoplasmic localization for both the GFP-NES1-SAR and GFP-NES2-SAR proteins (Figure 2C, panels 1, 2, 7 & 8). Thus, both the ESE-1 NES1 and NES2 sequences are sufficient to mediate nuclear export. Because NES motifs conforming to the (L/I)X2-4(L/I)X1-4(L/I)X(L/I) consensus sequence activate nuclear export by binding directly to the CRM1 nuclear exporter protein [24], we next tested the role of CRM1 in the nuclear export mediated by each ESE-1 NES motifs. MCF-12A cells transfected with the GFP-NES1-SAR or GFP-NES2-SAR constructs were treated with the CRM1-specific inhibitor leptomycin B [25], which resulted in the redistribution of GFP-NES1-SAR (Figure 2C, panels 3 & 4) and GFP-NES2-SAR (Figure 2C, panels 9 & 10), respectively, from the cytoplasm both to the nuclear and cytoplasmic compartments. This leptomycin B-induced inhibition of nuclear export reveals that both ESE-1 NES motifs function via a CRM1-dependent mechanism. The four conserved leucine/isoleucine residues characterizing the NES (L/I)X2-4(L/I)X1-4(L/I)X(L/I) sequence are known to play a crucial role in the function of this motif [26]. Thus, we next tested the functional importance of the conserved leucine/isoleucine residues in each ESE-1 NES by engineering two leucine/isoleucine-to-alanine mutations within the NES sequences of the GFP-NES1-SAR and GFP-NES2-SAR constructs (Figure 2B). NES1 was altered from LCNCALEELRL to LCNCAAEEARL (NES1Mut-SAR), and NES2 was altered from LWEFIRDILI to LWEFARDALI (NES2Mut-SAR). For both NES mutant plasmids, the GFP signal was diffusely nuclear and cytoplasmic (Figure 2C, panels 5, 6, 11 & 12), mimicking the GFP-NES1-SAR and GFP-NES2-SAR fluorescence patterns observed following leptomycin B treatment (Figure 2C, panels 3 & 9). These data demonstrate that the nuclear export function of each ESE-1 NES depends on conserved leucine/isoleucine residues within each of the NES sequences.

### Site-specific mutation of ESE-1 NES2 inhibits GFP-ESE-1-induced MCF-12A cell transformation

**Figure 3:**
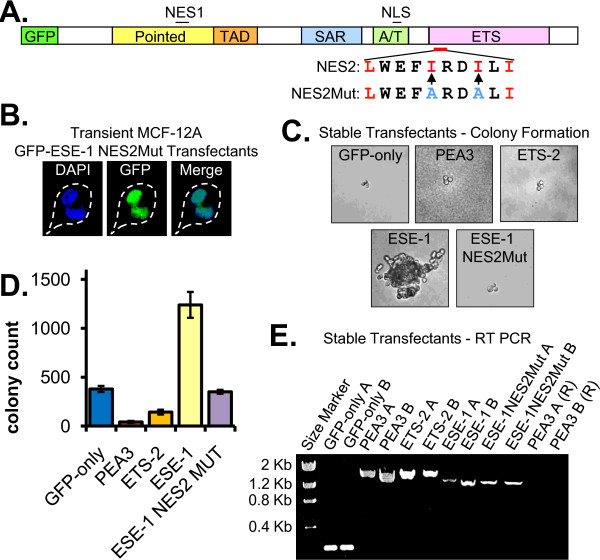
**Mutation of ESE-1 NES2 inhibits GFP-ESE-1-induced MCF-12A cell transformation**. **(A) **Diagram of the GFP-ESE-1 protein showing the two isoleucine to alanine point mutations engineered in the ESE-1 NES2 sequence to generate the GFP-ESE-1 NES2Mut construct. Black lines above the diagram - NES1 or NLS. Red line below the diagram - NES2 (wild type NES2 sequence expanded below). Red letters - critical leucine and isoleucine residues in NES2. Blue letters - the isoleucine to alanine point mutations engineered to generate NES2Mut. **(B) **Direct fluorescence imaging of MCF-12A cells transiently expressing GFP-ESE-1 NES2Mut. DAPI panel - nuclear DAPI florescence (blue). GFP panel - GFP fluorescence (green). Merge panel - an overlay of the DAPI and GFP fluorescence. **(C) **Anchorage-independent growth of pooled MCF-12A cell populations expressing GFP-only, GFP-PEA3, GFP-ETS-2, GFP-ESE-1 or GFP-ESE-1 NES2Mut. Stable transfectants were plated in soft agarose and typical colonies were photographed eight days later (40X). **(D) **Quantitation of anchorage-independent colony formation. Two independent, stably transfected MCF-12A populations for each construct (GFP-only, GFP-PEA3, GFP-ETS-2, GFP-ESE-1 and GFP-ESE-1 NES2Mut) were seeded in triplicate soft agarose cultures and colonies visible by 6X bright field microscopy were counted 21 days later. Bars show average numbers of colonies per plate +/- s.e.m. **(E) **RT-PCR analysis of mRNA expression of GFP fusion constructs in stably transfected MCF-12A cells. For each construct two independently generated stable populations (A and B) were analyzed: GFP-only populations (GFP-only A and B), GFP-PEA3 populations (PEA3 A and B), GFP-ETS-2 (ETS-2 A and B), GFP-ESE-1 (ESE-1 A and B), GFP-ESE-1 NES2Mut (ESE-1NES2MutA and B). PEA3 A (R) and PEA3 B (R) - RNAse treated samples.

Having shown that ESE-1 contains two separate, CRM1-dependent NES signals (Figure 2), we next sought to determine their role in the transforming function of full-length ESE-1 (Figure 3). We have previously reported that in-frame deletion of the ESE-1 Pointed domain, which contains NES1 (AA 102-112, Figure 3A), does not impair GFP-ESE-1-induced MCF-12A cell transformation [13]. Thus, the nuclear export function of NES1 is not required for the transforming function of GFP-ESE-1, since ESE-1-initiated transformation requires cytoplasmic localization, and inactivation of the critical NES signals should eliminate ESE-1 transforming activity. To test the role of NES2, we generated the same inactivating NES2 mutations as described for the GFP-NES2Mut-SAR construct (Figure 2B), but in the context of the full length ESE-1 protein (Figure 3A). As expected, this GFP-ESE-1 NES2Mut protein is exclusively nuclear in transiently transfected MCF-12A cells (Figure 3B). To test the effect of NES2 mutation on GFP-ESE-1-mediated transformation, we generated two independent stable MCF-12A transfectant populations for the GFP-ESE-1 NES2Mut construct, as well as for the GFP-ESE-1 and GFP-only constructs. In addition, because both the PEA-3 and ETS-2 ETS factors have been implicated in human breast cancer (although neither contains a SAR domain) we also fused GFP, in-frame, to the N-terminus of each of these ETS proteins and used these two fusions to test both their transforming potency and to control for nonspecific transforming effects of ETS protein expression in MCF-12A cells. Two independent MCF-12A stable cell populations were generated for each GFP-PEA3 and GFP-ETS-2 constructs. Subsequently, soft agar colony assays for all transfectant populations were performed in triplicate. Representative colonies in each culture were imaged at eight days (Figure 3C) and quantitated at 21 days (Figure 3D) post seeding. The GFP-only negative control did not yield multicellular colonies at eight days, whereas large multicellular colonies were formed by the GFP-ESE-1 positive control (Figure 3C). Further, the GFP-PEA3, GFP-ETS-2 and GFP-ESE-1 NES2Mut stable transfectants produced colonies similar to those observed in the GFP-only negative control. Colony quantitation for each stable transfectant revealed that the GFP-only negative control produced on average 379 colonies per plate and that the GFP-ESE-1 positive control formed 1239 colonies (Figure 3D). The GFP-PEA3 and GFP-ETS- 2 stable transfectants formed only 43 and 143 colonies, respectively (Figure 3D), suggesting that these two fusion proteins may exert a dominant negative effect on basal MCF-12A cell growth in soft agarose. Finally, stable GFP-ESE-1 NES2Mut expression resulted in only 350 colonies (Figure 3D). These data indicate that NES2 mutation abrogates GFP-ESE-1 transforming function in MCF-12A cells, confirming the colony imaging data shown in Figure 3C and also demonstrating that NES1 cannot compensate for lost NES2 function in full length ESE-1. Moreover, these findings indicate that neither PEA-3 nor ETS-2 possess transforming activity and that the nuclear export function of NES2 is essential for full-length ESE-1 transforming function in mammary epithelial cells. To confirm the expression of each GFP-ETS fusion construct in respective stable transfectants, we performed RT-PCR analysis and we sequenced the resulting PCR products for all stable cell populations described above. As shown in Figure 3E, these RT-PCR studies revealed that the two independently generated GFP-only stable populations (GFP-only A and GFP-only B) yielded only the expected 169-bp product. Similarly, only the expected 1624-bp GFP-PEA3-specific product was amplified from each GFP-PEA3 stable population (PEA3 A and PEA3 B), and each GFP-ETS-2 stable population demonstrated only the expected 1579-bp RT-PCR product (ETS-2 A and ETS-2 B). The ESE-1 A, ESE-1 B, ESE-1NES2Mut A and ESE-1NES2Mut B lanes, each representing a corresponding stable transfectant population, all exclusively demonstrated the same expected 1285-bp RT-PCR product. The presence of DNA contamination was assessed by treating total RNA from each stably transfected GFP-PEA3 pool with RNAse A (PEA3 A (R) and PEA3 B (R), respectively) prior to RT-PCR. Furthermore, DNA sequencing of these RT-PCR products demonstrated both the predicted in-frame GFP fusion and the absence of mutations in each case (data not shown).

### Stable expression of the GFP-NES1-SAR protein is sufficient to transform MCF-12A cells

**Figure 4:**
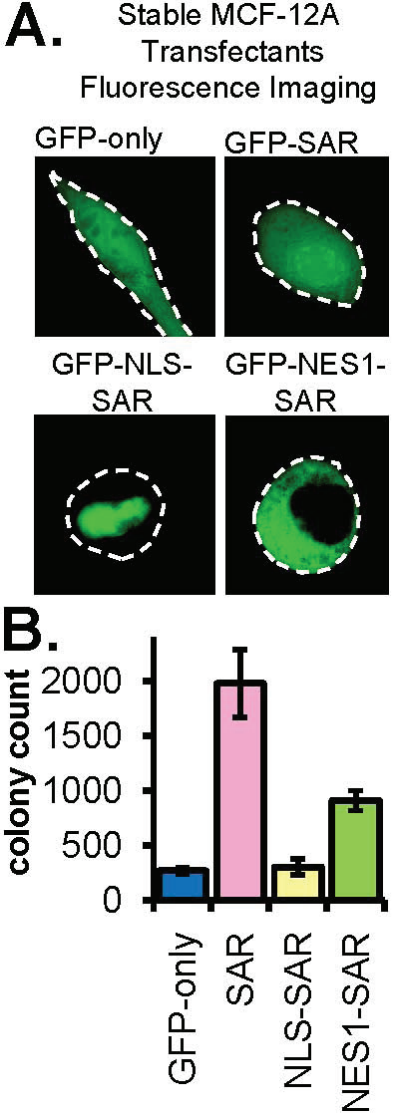
**Stable expression of the cytoplasmically restricted GFP-NES1-SAR protein is sufficient to transform MCF-12A Cells**. **(A) **Fluorescence imaging of stably expressed GFP-only, GFP-SAR, GFP-NLS-SAR and GFP-NES1-SAR in living MCF-12A cells. Green - GFP fluorescence. White dashed lines represent cell outlines. **(B) **Quantification of anchorage-independent colony formation of pooled MCF-12A cell populations stably expressing the GFP-only, GFP-SAR, GFP-NLS-SAR or GFP-NES1-SAR proteins. Two independent, stably transfected MCF-12A populations for each construct were seeded in triplicate soft agarose cultures and colonies visible by 6X bright field microscopy were counted 21 days later. Bars show average numbers of colonies per plate +/- s.e.m.

Using a GFP-fusion strategy similar to that described above, we have shown that the SAR domain of ESE-1 is both necessary and sufficient to mediate MCF-12A cell transformation and that enforced nuclear localization of the SAR domain abrogates this effect [13]. These data suggest that the SAR domain transforms MCF-12A cells via a cytoplasmic mechanism. Having generated GFP-NES-SAR fusion constructs whose expression is restricted to the cytoplasm (Figure 2), we used these reagents to directly test whether cytoplasmically restricted SAR protein is sufficient to initiate transformation. To this end, we generated two independent stable MCF-12A transfectant cell populations for GFP-NES1-SAR. As negative controls we generated stable MCF-12A transfectant populations for the GFP-only and GFP-NLS-SAR fusions, and as positive control we generated stable transfectants for GFP-SAR. Figure 4A shows representative subcellular GFP fluorescence patterns for MCF-12A cells stably expressing the GFP, GFP-SAR, GFP-NLS-SAR and GFP-NES1-SAR proteins. Note that while Figures 1 and 2 show GFP fluorescence patterns in transiently transfected MCF-12A, Figure 4A shows stable transfectants. As shown in Figure 4A, in each case, stable fusion protein localization is identical to that observed in transient transfectants. Specifically, GFP-only and GFP-SAR are both nuclear and cytoplasmic, the GFP-NLS-SAR is exclusively nuclear and stable GFP-NES1-SAR is exclusively cytoplasmic (Figure 4A). This restricted localization of GFP-SAR constructs is further corroborated in large-field images of transiently transfected MCF-12A and HeLa cells (Additional file 1). Of note, the selection of the GFP-NLS-SAR and GFP-NES1-SAR constructs for this experiment was arbitrary; any GFP-SAR fusion targeted to the nucleus (i.e. GFP-NLS1-SAR, GFP-NLS2-SAR, or GFP-NLS4-SAR, Figure 1C) or to the cytoplasm (GFP-NES2-SAR, Figure 2B) should function equivalently to the respective constructs chosen for analysis here (GFP-NLS-SAR and GFP-NES1-SAR). To test the transforming function of each stably expressed protein, each of the two independent stable MCF-12A transfectant cell populations were used to seed triplicate soft agarose cultures and colonies in each culture were quantitated at 21 days post seeding (Figure 4B). Quantitation studies revealed that the GFP-only and GFP-NLS-SAR negative control stable MCF-12A transfectants formed ~269 colonies and ~305 colonies, respectively (Figure 4B), demonstrating that GFP-NLS-SAR and the GFP-only are equivalently deficient in transforming function. In contrast, stable GFP-SAR and GFP-NES1-SAR expression produced ~1979 and ~1022 colonies, respectively (Figure 4B), revealing that both constructs transform cells, although NES-SAR shows 50% reduced colony formation. These data establish that cytoplasmically-restricted ESE-1 SAR domain is sufficient to transform MCF-12A cells.

### An intact SAR domain is required for optimal transforming activity

**Figure 5:**
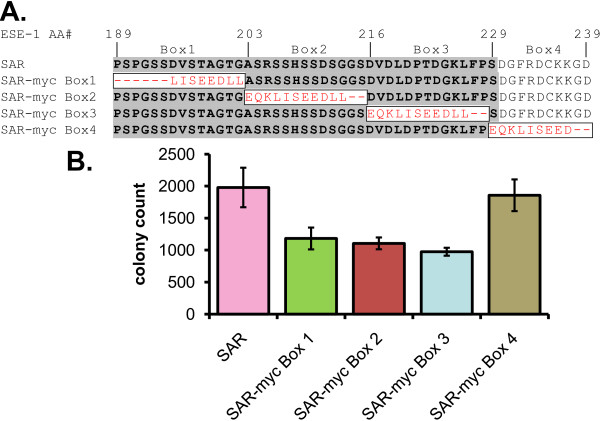
**An intact SAR domain is required for optimal transforming activity**. **(A) **Amino acid sequence of the SAR portion of GFP-SAR (SAR, top row) followed by sequences of GFP-SAR-myc Box 1 - Box 4 mutants, containing in-frame replacement of SAR domain amino acids 1-14 (SAR-myc Box 1, replaced AAs correspond to ESE-1 AAs 189-202), SAR amino acids 15-27 (SAR-myc Box 2, replaced AAs correspond to ESE-1 AAs 203-215), SAR amino acids 28-40 (SAR-myc Box 3, replaced AAs correspond to ESE-1 AAs 216-228), and amino acids 41-50, just distal to the SAR domain (SAR-myc Box 4, replaced AAs correspond to ESE-1 AAs 229-239). Black capital letters on gray background - SAR sequence. Black capital letters on white background - carboxy-terminal ESE-1 flanking sequence. Boxed red letters - myc epitope amino acids. Numbers above the SAR sequence indicate corresponding amino acid locations in the ESE-1 protein. **(B) **Quantification of anchorage-independent colony formation of pooled MCF-12A cell populations stably expressing GFP-SAR-myc Box 1 - Box 4 constructs (SAR-myc Box 1 - Box 4). Three independent, stably transfected MCF-12A populations for each construct were seeded in triplicate soft agarose cultures and colonies visible by 6X bright field microscopy were counted 21 days later. Colony numbers formed by GFP-SAR transfectants (SAR, purple bar), included here to facilitate comparison between SAR-myc Box 1-4 mutants and GFP-SAR control population, are reproduced from Figure 4C. Bars show average numbers of colonies per plate +/- s.e.m.

Having shown that the SAR domain is necessary and sufficient to initiate transformation of benign MECs [13], we next sought to fine map the subregions within the SAR domain that are essential for MEC transformation. To this end, we generated GFP-SAR mutants, in which we sequentially replaced 11-14 amino acid blocks of the SAR domain, in-frame, with 8-11 amino acid sequences of the Myc epitope, derived from the human c-myc protein, and we tested the ability of the resulting constructs to transform MCF-12A cells. Specifically, we replaced SAR amino acids 1-14 (SAR-myc Box 1, replaced AAs correspond to ESE-1 AAs 189-202), SAR amino acids 15-27 (SAR-myc Box 2, ESE-1 AAs 203-215), and SAR amino acids 28-40 (SAR-myc Box 3, ESE-1 AAs 216-228), each targeting sequential amino acid blocks of the 40-AA SAR domain (Figure 5A). As a control we also replaced amino acids 41-50, just distal to the SAR domain (SAR-myc Box 4, ESE-1 AAs 229-239), (Figure 5A). For each GFP-SAR mutant we generated three separate MCF-12A stable transfectant cell populations, and each stable transfectant population was then used to seed triplicate soft agarose cultures. Colony counts, performed 21 days post seeding, revealed that SAR-myc Box 1, SAR-myc Box 2 and SAR-myc Box 3 stable transfectants formed 1117, 1105, and 975 colonies, respectively, while SAR-myc Box 4 control transfectants formed 1823 colonies (Figure 5B). Thus, MCF-12A cells transfected with SAR-myc Box 1, SAR-myc Box 2 and SAR-myc Box 3 constructs formed 50% less colonies than cells transfected with either intact GFP-SAR (1987 colonies, Figure 4C & Figure 5B, purple bar) or control SAR-myc Box 4 transfectants. This result reveals that while SAR-myc Box 1, SAR-myc Box 2, and SAR-myc Box 3 mutants are capable of conferring the transformed phenotype to MCF-12A cells, their transforming activity is reduced by 50% compared to GFP-SAR, indicating that an intact SAR domain is required for the full transforming effect.

### The SAR domain contains the epitope for anti-ESE-1 mAb405

**Figure 6:**
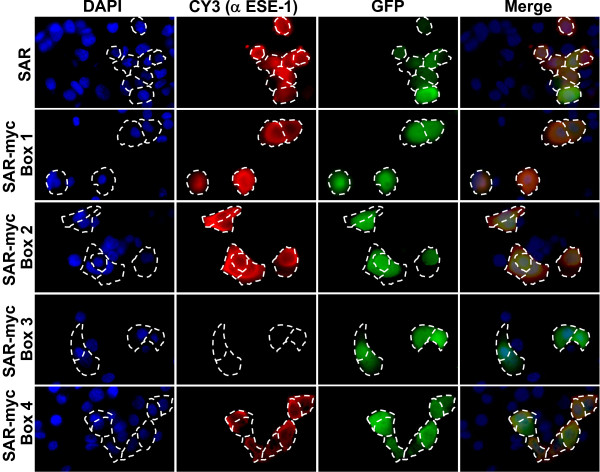
**The SAR domain contains the region that binds α-ESE-1 mAB405 antibody**. Immunofluorescence and direct fluorescence images of MCF-12A cells transiently transfected with GFP-SAR (SAR) and GFP-SAR-myc Box 1 - Box 4 mutant constructs (labeled SAR-myc Box 1 - SAR-myc Box 4, respectively). All cells were stained with anti-ESE-1 antibody mAB405 followed by secondary anti-mouse IgG conjugated to Cy3. DAPI - blue channel, DAPI fluorescence. Cy3 (αESE-1) - red channel, indirect immunofluorescence signal indicating binding of mAB405. GFP - green channel, direct fluorescence of GFP fusion protein. Merge - overlay of blue, red and green channels.

The ability of the SAR domain to initiate transformation via a cytoplasmic mechanism most likely requires SAR interaction with other proteins. To gain further insight into the mechanism of SAR domain action, we examined whether the SAR domain is surface exposed, and therefore has the potential to mediate protein-protein interactions. As a test of whether the SAR domain is surface exposed, we generated monoclonal antibodies to an antigen spanning ESE-1 amino acids 129-259, containing the TAD through AT hook domains of ESE-1 and tested whether any of these antibodies could recognize the SAR domain. Using the panel of anti-ESE-1 monoclonal antibodies in Western blot analysis, we found that mAb405 recognized the SAR domain with high affinity (data not shown). To map the precise region of SAR that interacts with mAB405 antibody we took advantage of the four aforementioned SAR-myc box mutants, and performed immunofluorescence analysis of MCF-12A cells transfected with these constructs (Figure 6). As shown in Figure 6, monoclonal antibody mAB405 detected intact GFP-SAR, as well as mutants SAR-myc Box 1, Box 2, and Box 4, revealing that amino acids 189-215 of the SAR domain and amino acids 229-239 just distal to the SAR domain do not contain the mAB405 epitope. However, the immunofluorescence signal was completely lost with the Box 3 mutant spanning amino acids 216-228 of the SAR domain, indicating that this 13-AA SAR fragment contains the mAB405 epitope. This finding identifies ESE-1 amino acids 216-228 as an antibody-accessible region, which is likely to be surface-exposed and readily accessible for protein-protein interactions.

## Discussion

ETS family proteins have been shown to function in the nucleus as regulators of gene transcription [1-4]. However, despite previous documentation of ESE-1 transcription factor function [12,13,20,27-30], we have proposed a novel nontranscriptional, cytoplasmic model whereby ESE-1, functioning via its SAR domain, initiates mammary epithelial cell transformation [13]. For ESE-1 to mediate transformation from a cytoplasmic location, ESE-1 must contain a functional nuclear export sequence. In this report, we used molecular and pharmacological methods to define functional NLS and NES sequences within human ESE-1 and to characterize the critical role of nuclear export of ESE-1 in its transforming function. Furthermore, we demonstrated that cytoplasmically-restricted SAR domain is sufficient to initiate MEC transformation and that full transforming activity requires an intact SAR domain.

ESE-1 has been documented to operate as a nuclear activator of promoter function in transient transfection reporter assays [28-30]. Indeed, transient transfection of GFP-ESE-1 into several different cell lines, including HeLa cervical carcinoma and T47D and SKBR-3 breast cancer cells, demonstrates nuclear localization of this fusion protein (data not shown). In this report we use progressive truncations in GFP-fusion gain-of-function studies to map ESE-1 nuclear localizing activity to a basic, six AA sequence (^242^HGKRKR^247^) located within the A/T Hook domain, but outside of the ESE-1 DBD (Figures 1A, B, &1C). We confirmed that the DBD does not contain an NLS sequence required for nuclear localization of ESE-1, using a loss-of-function deletion study of the ESE-1 DBD, demonstrating that DBD deletion does not impair ESE-1 nuclear import (Figure 1E). Furthermore, we have previously reported that in-frame deletion of the ESE-1 A/T Hook domain (AAs 236-267), which includes the functional ESE-1 NLS identified here, completely inhibits ESE-1 nuclear import [13]. Indeed, Elf3, the murine ortholog of ESE-1, has been shown to contain a functional NLS located at an equivalent position and, in contrast to ESE-1, an additional NLS in its DBD [21]. Interestingly, while both Elf3 NLS motifs function autonomously in fluorescent protein-fusion assays, they appear to target to different subnuclear regions. However, since neither of the Elf3 NLS motifs has been individually mutated or deleted in the context of full-length Elf3, the requirement of either NLS in Elf3 nuclear targeting remains unknown. Nevertheless, our data confirm that the functional ESE-1 NLS resides within the A/T hook domain and that ESE-1 DBD is neither necessary nor sufficient to mediate ESE-1 nuclear localization. This finding is surprising in light of previous reports demonstrating an essential role for the highly conserved ETS DBD in ETS factor nuclear localization [17-19]. Finally, amino acid comparison analyses performed by us and others [31] reveal that the ESE-1 NLS discovered here is not present in any other ETS factor, including other members of the ESE subfamily.

Extensive evidence supports nuclear-cytoplasmic shuttling as a regulatory mechanism for ETS protein function [32-35]. A common regulatory mechanism involves MAPK signaling cascades, which trigger nuclear export of ETS repressors such as NET (new ETS), YAN, ERF (ETS-2 Repressor Factor) and TEL (Translocation ETS Leukemia) and thus release ETS-mediated gene repression. For example, the ETS DBD of the ternary complex factor NET contains a functional, CRM1- dependent NES (^7^LWQFLLQLLL^16^) that appears to be highly-conserved within the DBDs of most ETS proteins [32], including ESE-1 (i.e. NES2: ^275^LWEFIRDILI^284^). Activation of the c-Jun N terminal kinase kinase (JNKK) pathway mediates nuclear exclusion of NET, relieving transcriptional repression induced by NET. Moreover, site-specific mutation of the NET NES traps NET in the nucleus, resulting in increased NET repressor function [32]. These data point to a crucial regulatory role for the NET NES.

In this report, we identify two ESE-1 NES signals, NES1 and NES2, but we demonstrate that only one, NES2, plays a critical role in the nuclear export and transforming function of intact ESE-1 protein. NES1 is located in the ESE-1 Pointed domain but appears to mediate nuclear export, in a CRM1-dependent manner, only when outside of the context of full length ESE-1 protein (Figure 2C). In addition, comparative analysis of ETS factors Pointed domain sequences reveals that most other ETS factors, including ESE-2 and ESE-3, do not conserve the NES1 motif. In contrast, NES2 appears to be well conserved in the DBD region of most ETS proteins, suggesting a conserved function of this motif in the ETS family (analysis not shown). Here we show that inactivating mutations in the ESE-1 NES2 completely inhibit GFP-ESE-1 transforming function (Figures 3C &1D), indicating that GFP-ESE-1 nuclear export plays an essential role in GFP-ESE-1-mediated transformation. An alternative to this conclusion is that mutation of DBD-embedded NES2 disrupts ETS DBD-DNA binding and that it is this disruption, rather than the inhibition of NES2 function, that impairs GFP-ESE-1 transforming activity. However, crystallographic structural data for the DNA-bound ESE-1 DBD indicate that NES2 is localized to a DBD subregion that does not make direct contact with target DNA, except for leucine 275 [36]. This finding is consistent with our previously published data showing that the domains of ESE-1 that are required for transcription factor function (e.g. the ESE-1 TAD) are not necessary to initiate transformation in benign MECs, whereas the SAR domain alone is sufficient in this type of transformation assay (Figure 4) [13]. As noted above, the ESE-1 NES2 is similar in sequence and location to the functional MAPK-regulated NES motifs in NET and ERF. However, we have been unable to identify any specific kinase(s) that regulate ESE-1 subcellular localization. Specifically, co-transfection studies using constitutively active forms of JNK, MAPK, ERK and v-SRC protein kinases revealed that none of these kinases enhanced cytoplasmic shuttling of transiently co-expressed nuclear GFP-ESE-1 (data not shown). Taken together, our data suggest that basal ESE-1 subcellular localization represents the summed influences of NES and NLS functions.

**Figure 7:**
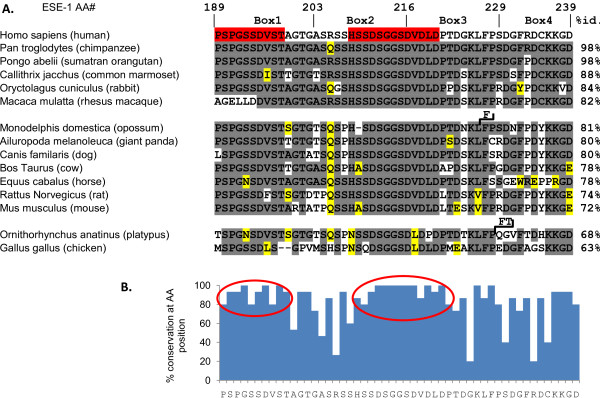
**Alignment of SAR sequences from 15 species retrieved from the NCBI database**. **(A) **Amino acid sequences of SAR domains from 15 species found in NCBI database http://blast.ncbi.nlm.nih.gov ordered according to percent identity to human SAR amino acid sequence. Gray background - AAs identical to human sequence. Yellow background - conservative substitutions. Red background - conserved regions. Black angled lines - insertions. %id. - percent identity to human protein sequence. Conservative substitutions and gaps were regarded as non-identical AAs for calculations of percent identity. **(B) **Percent conservation at each AA position between human and the remaining 14 species listed in (A). Conservative substitutions and gaps were considered non-identical amino acids for the purpose of calculation of percent conservation. Red ellipses - conserved subregions of SAR.

A key finding in this report is that the ESE-1 SAR domain alone, as GFP-NES-SAR, can be stably and specifically targeted to the cytoplasm in MCF-12A cells and that this cytoplasmic localization is sufficient to initiate MEC transformation (Figure 4). The decreased transformation potency of GFP-NES-SAR vs. GFP-SAR observed in our study is most likely due to the differential levels of expression of GFP-NES-SAR vs. GFP-SAR. However, another possible explanation of this result is that the nuclear fraction of SAR contributes to the transformation, even though it is insufficient to evoke any transformation effect by itself. Furthermore, all of the data to date point to the likely requirement that the SAR domain interacts with other protein(s) to initiate transformation. Supporting this notion are the observations that amino acids 216-228 are accessible to mAB405 (Figure 6), and that Pak-1 phosphorylates serine 207, with β-TrCP ubiquitinating the S207-dephosphorylated form and targeting it for proteosome-mediated degradation [37]. Indeed, the report by Manavathi et al. [37] provided important insights into the mechanisms of transformation initiation in benign MCF-12A MECs by ESE-1, revealing that Pak-1 mediated phosphorylation of serine 207 within the SAR domain, results in increased protein stability and increased transformation potency of ESE-1 [37]. Of note, site-specific mutation of serine 207 to alanine resulted in 50% loss of soft agar colony formation, which is consistent with our SAR-myc Box 2 data, also showing 50% reduction (Figure 5). However, mutation of Box 1 and Box 3 which span the amino- and carboxy- terminal regions of the SAR domain, respectively, also resulted in about 50% loss of transformation activity (Figure 5), suggesting that an intact three-dimensional structure of the SAR domain is required for optimal transformation potency. One caveat in this study was that myc Boxes 1, 2, and 3 all contain the sequence LISEEDLL, while in myc Box 4 the two terminal "LL" amino acids are missing. This may lead to an alternative interpretation that the LISEEDLL motif within the myc sequence functions as an active inhibitor of transformation, and that the two terminal "LL" amino acids are required for inhibitor function. To gain further insight of the key structure-function aspects of the SAR domain, we performed a phylogenetic analysis of SAR domain protein sequences derived from fifteen different species, of which fourteen are mammalian, in order to identify the most conserved regions (Figure 7). This comparison revealed that the SAR domain is found only in ESE-1 orthologs and in no other proteins in the NCBI database (not shown). However, the SAR domain is highly conserved among mammals, with a clear reduction in conservation in the chicken SAR sequence (Figure 7). Furthermore, there appear to be two highly conserved subregions: amino acids 189-198 and amino acids 208-220. While the conserved amino terminal region appears not to contain any known functional motifs, the region containing amino acids 208-220 coincides with the PEST sequence, ^209^SSDSGGSDVD^218 ^identified previously [37], and a highly conserved putative CKII phosphorylation site, ^217^SDVD^220^. However, S207 is mutated to proline in 6 out of 15 species in the database.

## Conclusions

Our data precisely define NLS and NES signals in the human version of ESE-1 that play a pivotal role in regulating its subcellular localization and its ensuing transforming function. Furthermore, we report that transformation of human MECs requires an intact SAR domain that can be targeted exclusively to the cytoplasm, and that the SAR motif is accessible for protein and/or ligand interactions. This report is important, since it provides critical mechanistic details of ESE-1 function, and it significantly expands our understanding of the role of ETS factors in mammary cell transformation.

## Methods

### Mammalian cell culture

All cell lines were acquired from the American Type Culture Collection (Manassas, VA) and were maintained as described in [13].

### GFP fusion vectors

All GFP-SAR fusion constructs were generated using the previously described PCR-based strategy [13] and subcloned into the pEGFP-C3 parental expression plasmid (Invitrogen Inc., Carlsbad, CA). Specific sense primers for GFP-SAR fusions were: 5'- cgg**gaattc**atcccaagcacgggaagcggaaacgaTCCCCTGGCAGCTCTG (GFP-NLS1-SAR); 5'- ccg**gaattc**at*aagaagggggatcccaagcacgggaagcggaaacga*TCCCCTGGCAGC (GFP-NLS2-SAR); 5'- cgg**gaattc**atcgaaagctgagcaaagagtactgggactgtctcgagggcaagaagagcaagcacTCCCCTGGCAGCTCTGAC (GFP-NLS3-SAR); 5'- cgg**gaattc**ataagcacgggaagcggaaacgaTCCCCTGGCAGCTCTG (GFP-NLS4-SAR); 5'- cgg**gaattc**atcacgggaagcggaa acgaTCCCCTGGCAGCTCTG (GFP-NLS5-SAR); 5'- cgg**gaattc**atggccaaaagaaaaagaacagcaacTCCCCTGGCAGCTCTG (GFP-NLS6-SAR). 5'- cgg**gaattc**atctctgcaattgtgcccttgaggagctgcgtctgTCCCCTGGCAGCTCTG (GFP-NES1-SAR); 5'- cgg**gaattc**atctctgcaattgtgccgccgaggaggcccgtctgTCCCCTGGCAGCTCTG (GFP-NES1Mut-SAR); 5'- cgg**gaattc**atctgtgggagttcatccgggacatcctcatcTCCCCTGGCAGC (GFP-NES2-SAR); 5'- cgg**gaattc**atctgtgggagttcgcccgggacgccctcatcTCCCCTGGCAGC (GFP-NES2Mut-SAR); Bold sequences show *EcoR*I restriction sites, capital letters represent SAR coding sequence and NES/NLS sequences are underlined. Generation of GFP-ESE-1ΔDBD and GFP-ESE-1 NES2Mut was accomplished using a two-step PCR overlap extension strategy [38,39]. In both cases, the 5' ESE-1 sense primer [13] was used with the following antisense primers to generate the 5' segment of each construct, respectively: 5'-GGCAAAAACTCAAGCGGCTGGAAG (GFP-ESE-1ΔDBD antisense) and 5'-GATGAGGGCGTCCCGGGCGAACTCCCACAG (ESE-1 NES2Mut antisense). To generate 3' overlap segments, the 3' ESE-1 antisense primer [13] was used with the following respective sense primers: 5'-AGTTTTTGCCGTGGGTGCCTCTG (GFP-ESE-1ΔDBD sense) and 5'- CTGTGGGAGTTCGCCCGGGACGCCCTCATCCACCCGGAGCTCAACGAG (ESE-1 NES2Mut sense). The resulting PCR overlap extension products were ligated into the pEGFP-C3 plasmid as described previously [13]. Similar PCR strategy, followed by ligation into pEGFP-C3 plasmid, was used to generate GFP-SAR-myc Box 2 and GFP-SAR-myc Box 3 constructs. In both cases, 5' SAR sense primer [13] was used with the following respective antisense primers to generate the 5' segments of each construct: 5'- *gtcttcctcgctgatcagtttctgctc*ACCAGTCCCTGCGGTGGAG (GFP-SAR-myc Box 2 antisense), 5'- *gtcttcctcgctgatcagtttctgc*TCACTTCCACCGGAGTCTGAGG (GFP-SAR-myc Box 3 antisense). Whereas, the 3' SAR antisense primer [13] was used with the following respective sense primers to generate the 3' segment of each construct: 5'- *ctgatcagcgaggaagacctgttg*GACGTGGACCTGGATCCCACTG (GFP-SAR-myc Box 2 sense), 5'- *ctgatcagcgaggaagacctgttg*AGCGATGGTTTTCGTGACTGC (GFP-SAR-myc Box 3 sense). To produce the GFP-SAR-myc Box 1 construct, the following sense primer was used in a PCR with the 3' SAR antisense primer [13]: 5'- *ccggaattcatctgatcagcgaggaagacctgttg*GCTTCTCGGAGCTCCCACTCC. Similarly, the GFP-SAR-myc Box 4 sequence was amplified using the following antisense primer in a PCR with the 5' SAR sense primer [13]: 5'- *ccggaattcatgtcttcctcgctgatcagtttctgctc*GGGGAAGAGCTTGCCATC. Both sequences were ligated into the pEGFP-C3 EcoR I site, to produce the GFP-SAR-myc Box 1 and GFP-SAR-myc Box 4 constructs, respectively. For each primer used in generation of GFP-SAR-myc Box mutants, capital letters show SAR domain coding sequence, and italicized text shows myc epitope sequence. To generate the pEGFP-PEA3 and pEGFP-ETS-2 expression plasmids, the full-length human PEA3 and ETS-2 coding sequences were amplified by RT-PCR from T47D human breast cell line whole cell RNA. The respective primer pairs used in these amplifications were as follows: 5'-cg**agatct**ccg**gaattc**atATGGAGCGGAGGATGAAAGCCGG vs. 5'-cg**agatct**ccg**gaattc**atCTAGTAAGAGTAGCCACCCTTG (PEA3) and 5'-cg**agatct**ccg**gaattc**atATGAATGATTTCGGAATCAAG vs. 5'-cg**agatct**ccg**gaattc**atTCAGTCCTTCGTGTCGGGC (ETS-2). In each case, restriction sites are in bold, and start and stop codons are underlined. Each full-length coding sequence was then ligated into the pEGFP-C3 plasmid as described [13]. The absence of mutations in each expression construct was confirmed by DNA sequencing.

### Fluorescence microscopy

MCF-12A cells were transfected with GFP-fusion expression plasmids and plated as described previously [13]. Alternatively, stable MCF-12A transfectants were plated directly onto glass coverslips for confocal microscopy. For nuclear staining, some cover slips were stained with 300 nM 4',6-diamidino-2-phenylindole (DAPI) [13]. In addition, some coverslips were incubated for 15 minutes at 37°C in PBS containing 10 ng/ml leptomycin B (Sigma-Aldrich, Inc., St. Louis, MO). Cell imaging and image acquisition were performed as described previously [13].

### Stable cell lines

Stable MCF-12A cell expression of each GFP fusion protein was obtained as described in [13] and two or three independent stable transfectant populations were generated for each expression plasmid.

### Soft agarose assays

Triplicate soft agarose cultures were prepared for each stable MCF-12A transfectant population, as described in [13]. Each experiment was repeated as noted in the text. Representative colonies were imaged and quantitated as described in [13].

### RT-PCR

Whole cell RNA was prepared from individual stable transfectant populations using an RNA STAT-60 kit (Tel-test, Inc., Friendswood, TX). GFP fusion transcripts in each RNA sample were identified using a sense primer directed against a terminal portion of the GFP open-reading frame and an antisense primer specific for a transcribed but untranslated sequence immediately downstream of the DNA insertion site in the pEGFP-C3 plasmid. The Omniscript™ RT kit (Qiagen) was used for reverse transcription as described in [13]. Some RNA samples were treated with RNAse A (Five Prime Three Prime, Inc., Boulder, CO) prior to reverse transcription. All RT-PCRs were analyzed by 1% agarose gel electrophoresis.

### Immunofluorescence

Cells were plated directly onto glass cover slips in a 12-well tissue culture plate and transfected with GFP-SAR constructs using Effectene (Qiagen). Two days post-plating, cells were fixed with 2% paraformaldehyde for 20-25 min at room temperature and washed with phosphate-buffered saline (PBS). Subsequently, cells were permeabilized with 0.5% Triton X-100 in PBS for 10 minutes, followed by three washes in 100 mM glycine in PBS. Permeabilized cells were blocked in blocking buffer containing 0.5% Tween-20, 10% goat serum in PBS for 1-2 h. Cells were incubated with anti-ESE-1 monoclonal antibody mAB405 [16] diluted 1:500 in the blocking buffer overnight at 4°C. After washes, cells were incubated for 1 h with Cy3-conjugated donkey anti-mouse IgG secondary antibody (Jackson Immunoresearch). Nuclei were counterstained using 300 nM DAPI (Invitrogen, #D-1306).

### Alignments of SAR protein sequences

Amino acid sequences were retrieved from non-redundant protein sequences NCBI database using BLASTP 2.2.24+ program and the human SAR sequence (ESE-1 AAs 189-239) as a query (http://blast.ncbi.nlm.nih.gov/, [40]). For the purpose of calculation of percent identity to human sequence, conservative substitutions and gaps were considered as non-identical amino acids. When there was an insertion, percent identity was calculated with the number of amino acids in the longer protein as the denominator. For the purpose of calculation of percent conservation at a given AA position between human and the remaining species, conservative substitutions and gaps were considered as non-identical amino acids, while insertions were excluded from analysis.

## Abbreviations

NLS: (Nuclear Localization Sequence); NES: (Nuclear Export Sequence); SAR domain: (Serine and Aspartic acid Rich domain); MEC: (mammary epithelial cell)

## Competing interests

The authors declare that they have no competing interests.

## Authors' contributions

JDP designed and performed experiments, analyzed the data and wrote the manuscript. JMP analyzed and interpreted the data, wrote the manuscript and performed SAR sequence alignments. JJT and DMW generated the mAB405 antibody. AGH conceived and directed the research and participated in writing of the manuscript. All authors read and approved the final manuscript.

## Supplementary Material

Additional file 1 **Supplemental Figure 1**. Large-field fluorescent images of MCF-12A cells and HeLa cells transiently transfected with GFP alone, GFP-SAR, GFP-NES-SAR, or GFP-NLS-SAR. Click here for file
