# Supplementary material for: Mapping of ESE-1 subdomains required to initiate mammary epithelial cell transformation via a cytoplasmic mechanism
Source: Mol Cancer. 2011 Aug 28;10:103. doi: 10.1186/1476-4598-10-103 (PMC3183030; doi:10.1186/1476-4598-10-103)
Supplement: Additional file 1 — Supplemental Figure 1. Large-field fluorescent images of MCF-12A cells and HeLa cells transiently transfected with GFP alone, GFP-SAR, GFP-NES-SAR, or GFP-NLS-SAR. [file 1476-4598-10-103-S1.PDF]

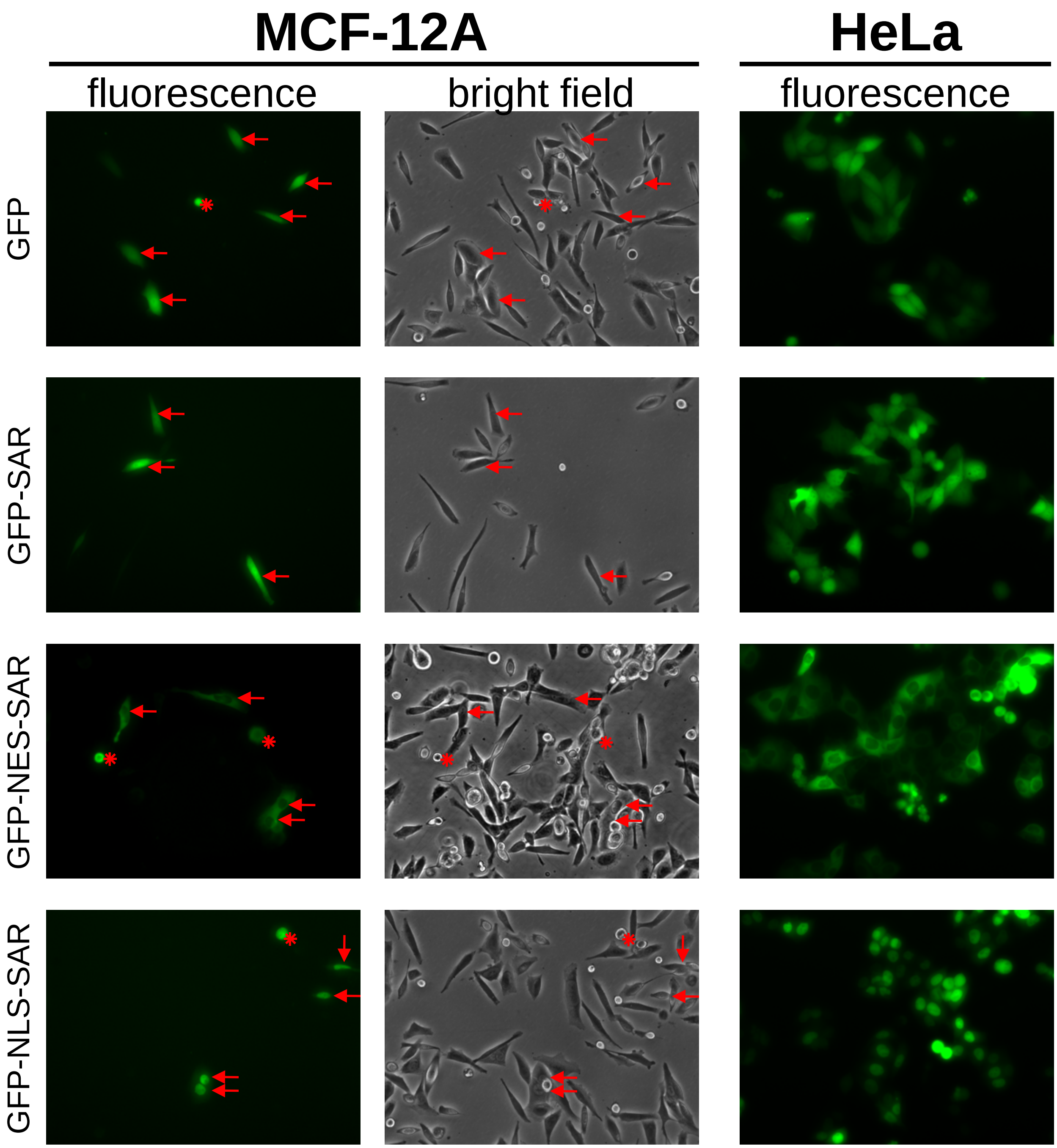

Supplemental Figure 1. MCF-12A cells and HeLa cells transiently transfected with GFP alone, GFP-SAR, GFP-NES-SAR, or GFP-NLS-SAR. Left column: Direct fluorescence imaging of MCF-12A cells transfected with GFP-SAR constructs, as indicated. Arrows – transfected cells, asterisks – cells that rounded up. Middle column: Corresponding brightfield images of transfected MCF-12A cells. Right column: Direct fluorescence imaging of HeLa cells transfected with GFP-SAR constructs, as indicated.
